# The Role of Sensitization to Allergen in Asthma Prediction and Prevention

**DOI:** 10.3389/fped.2017.00166

**Published:** 2017-07-31

**Authors:** Maria Moustaki, Ioanna Loukou, Sophia Tsabouri, Konstantinos Douros

**Affiliations:** ^1^Cystic Fibrosis Unit, “Aghia Sophia” Children’s Hospital, Athens, Greece; ^2^Department of Paediatrics, Child Health Department, University of Ioannina School of Medicine, Ioannina, Greece; ^3^Pediatric Allergy and Respiratory Unit, 3rd Department of Pediatrics, “Attikon” Hospital, University of Athens School of Medicine, Athens, Greece

**Keywords:** allergy, asthma, atopy, prediction, prevalence, prevention, sensitization, wheezing

## Abstract

The burden of asthma in childhood is considerable worldwide, although some populations are much more affected than others. Many attempts have been made by different investigators to identify the factors that could predict asthma development or persistence in childhood. In this review, the relation between atopic sensitization as an indicator of allergy and asthma in childhood will be discussed. Cross sectional studies, carried out in different countries, failed to show any firm correlation between asthma and atopic sensitization. Birth cohort mainly of infants at high risk for asthma and case–control studies showed that atopic sensitization was a risk factor for current asthma in children older than 6 years. In general, clear relations are observed mostly in affluent Western countries, whereas in less affluent countries, the picture is more heterogeneous. For the prediction of asthma development or persistence in school age children, other prerequisites should also be fulfilled such as family history of asthma and wheezing episodes at preschool age. Despite the conductance of different studies regarding the potential role of allergen avoidance for the primary prevention of childhood asthma, it does not seem that this approach is of benefit for primary prevention purposes. However, the identification of children at risk for asthma is of benefit as these subjects could be provided with the best management practices and with the appropriate secondary prevention measures.

## Introduction

Asthma is a very common disease in childhood. In certain countries, such as the USA, it has been estimated that it is the most frequently encountered chronic condition in childhood (1). Asthma affects adults too, but in the majority of cases, the origins of the disease are in the preschool age (2). Childhood asthma is a heterogeneous clinical disorder that includes different phenotypes at different ages (3). This heterogeneity was lucidly illustrated in the Tucson birth cohort study where almost 50% of children had at least one episode of wheeze until the age of 6 years. In 20% of these children, wheezing was restricted to the first 3 years of life (transient wheezers), in 15%, wheezing appeared after the third year of age (late-onset wheezers) and, in almost 15%, wheezing was present both before 3 years, and it lasted at least until the sixth years of age (persistent wheezers) (4).

From these results, we can surmise that children with wheezing at 6 years of age are unlikely to share the same risk factors with those who never wheezed or who have outgrown wheezing episodes by the age of 6 years. Similar, though not identical, phenotypes of preschool wheezing were also identified by other epidemiological studies (5, 6). Their results corroborated that only a subset of children with preschool wheezing will have further episodes by the age of 6 years. The above highlights the difficulties in predicting which of the preschool wheezers will eventually develop asthma as the majority will outgrow wheezing episodes by the age of 6 years. Furthermore, Saglani et al. (7) showed that eosinophilic inflammation, which is a characteristic of asthma in older children and adults, was not present in infants with recurrent wheezing and/or cough episodes even in the presence of reversible airflow obstruction. The lack of evidence for any underlying asthmatic-type inflammation and the poor agreement on definitions of different phenotypes of preschool wheezing urged the ERS Task Force in 2008 (8) to suggest not using the term asthma for preschool wheezers. Instead, they proposed the terms episodic (viral) wheeze for children who wheeze intermittently and are well between episodes, and multiple-trigger wheeze for those who wheeze both during and outside discrete episodes. These phenotypes may, however, change over time (9) and, therefore, their validity has been questioned (10).

However, regardless of what classification is used for preschool wheezers, the real challenge is to recognize the risk factors that predispose to the continuation of asthma symptoms in school-aged children and adolescents. The identification of these risk factors may allow the implementation of effective preventive and therapeutic strategies, to the extent that these factors are modifiable.

Atopy was traditionally considered a risk factor for the development of asthma; allergic march (or atopic march) is a term that has been used for many years to describe the hypothesis of progression from eczema in infancy to rhinitis and asthma in older children (11). According to the most recent definition of World Health Organization, the term “refers to the natural history of atopic manifestations, which is characterized by a typical sequence of immunoglobulin E (IgE) antibody responses and clinical symptoms, which may appear early in life, persist over years or decades, and often remit spontaneously with age” (12). Accordingly, it is considered that a substantial proportion of asthmatic patients have allergic asthma with an underlying IgE-mediated hypersensitivity mechanism and it is, therefore, called IgE-mediated allergic asthma (13). Based on this, it would be plausible to hypothesize that the identification of allergic children could lead to the identification of those at high risk for the development of asthma. Grounded on this hypothesis, many epidemiological studies have investigated whether allergy or atopy was a risk factor for asthma in childhood using either clinical surrogates such as eczema, or biomarkers such as eosinophilia, total IgE, or specific IgE antibodies to certain allergens. Other studies have tried to investigate the relationship between asthma and atopy and to clarify to what extent asthma in children can be attributed to atopy, through the parallel variation of both conditions’ prevalence in different populations (14).

The aim of this review is to present the existing data on the relation of atopic sensitization—and its use as an indicator—with asthma in school-aged children, and also the role of allergen exposure for primary prevention of childhood asthma.

## Prevalence of Asthma and Atopy in Cross-Sectional Studies Worldwide

The International Study of Asthma and Allergies in Childhood (ISAAC) revealed considerable variability of asthma prevalence among different countries and different regions, and sometimes among different areas of the same country (15, 16).

In 1999, Pearce et al. (17) summarized the studies that had evaluated the prevalence of asthma and atopy in children. They reviewed seven such studies from different populations and they concluded that most of them failed to demonstrate an association between asthma and atopy prevalence. Similarly, two other groups summarized the findings of the studies that compared asthma and atopy prevalence in childhood over different time periods and observed that there was either an increase of asthma over time without; however, a parallel increase in atopy (18, 19) or, that asthma prevalence was unchanged but the atopy prevalence was increasing.

Ronchetti et al. (20, 21) conducted two systematic reviews, and their main findings were the absence of correlation between the prevalence of asthma and the prevalence of atopy, and the existence of a strong correlation between atopy in asthmatic and atopy in the non-asthmatic children; the above imply that the prevalence of atopy in asthma depends on some environmental factors that simultaneously induce atopy in asthmatic and non-asthmatic individuals.

The role of atopic sensitization in the worldwide variation of asthma prevalence was also explored by Weinmayr et al. (22) who conducted cross-sectional studies in 32 centers from 22 countries worldwide in children aged 8–12 years following the ISAAC protocol. They found that current wheeze prevalence rates were not associated with the respective rates of atopic sensitization. However, the fraction of current wheeze attributable to atopic sensitization, as this was determined by skin prick test (SPT) reactivity, was higher in affluent compared to the non-affluent countries (40.7 and 20.3%, respectively). They also observed that this fraction was strongly correlated with the gross national income.

The above data (22) indicate that the potential link between asthma and atopic sensitization differs between countries and areas; as previous studies have already shown, strong relations are observed mostly in affluent Western countries (23–26), whereas in less affluent countries, the picture is more heterogeneous picture (24, 25, 27–29).

## Atopy as a Risk Factor for Childhood Asthma in Cohort and Case–Control Studies

Many studies have been designed and conducted with the aim to investigate the potential risk factors for the presence of asthma in childhood. Atopic sensitization is among the most commonly investigated risk factors for asthma in childhood. Table 1 depicts the characteristics of relevant studies that have been published since 2001. These studies were identified through PubMed searching, using the following terms: allergy, atopy, SPT, IgE, RAST, sensitization, children, asthma, and prediction.

**Table 1:** Studies that investigated atopic sensitization as a risk factor for the development of asthma in childhood.

No.	Reference	Study population	Indicator of atopic sensitization	Age of asthma diagnosis	Measured associations
1	Alduraywish et al. (30)	Two independent birth cohorts (1) the high risk MACS cohort and (2) the population-based LISAplus cohort	(1) MACS cohort: SPTs to food and inhalant allergens at 6, 12, and 24 months (2) LISApluscohort: sIgE to food and inhalant allergens at 2 years	10–12 years	The strongest effect on asthma risk was found in both cohorts, in subjects with co-sensitization to food allergens and aeroallergens
2	Boersma et al. (31)	166 children who visited a hospital with wheezing at the age 12–48 months	sIgE antibodies to inhalants allergens at the age of 12–48 months	At least 6 years	Sensitization to inhalant allergens has a positive predictive value of 86% for asthma. It remained a strong predictor for asthma even in multivariate analysis model
3	Anderson et al. (32)	Birth cohort, 289 newborns at high risk for asthma	sIgE antibodies to inhalant allergens at the age of 2, 6, and 11 years	6 and 11 years	Sensitization to aeroallergen at 2 years triples the risk of asthma at 6 years and at 11 years
4	Gabet et al. (33)	Birth cohort, 3,860 full term healthy singletons	sIgE antibodies to food and inhalant allergens at the age of 18 months	6 years	Current symptoms of asthma were significantly more frequent in children who were mono- or pauci-sensitized or multi-sensitized
5	Amin et al. (34)	Birth cohort, 762 newborns with a parent with a positive SPT to at least 1/15 aeroallergen, living either <400 m or >1,500 m from a major road	SPTs to aeroallergens, cow’s milk and hen’s egg at the age of 1, 2, 3, 4, and 7 years	7 years	Sensitization to >1 aeroallergen at 12 months of age or at 3 years was more frequent among children with asthma at 7 years. The same was true for sensitization to egg, but not for sensitization to cow’s milk
6	Rø et al. (35)	Subpopulation of a birth cohort, 668 children evaluated at 2 years of age from the PACT study birth cohort	sIgE antibodies and SPT to nine allergens at 2 years of age	6 years	Positive sIgE was associated with a significantly increased risk for asthma at the age of 6 years in the unadjusted for confounders model
7	van der Mark et al. (36)	A cohort of 771 children, aged 1–5 years, who visited primary care clinics during the preceding 12 months with complaints of recurrent coughing, wheezing, and/or shortness of breath	sIgE antibodies to dog, cat, HDM	6 years	Positive sIgE doubled the risk for asthma diagnosis at the age of 6 years
8	Stoltz et al. (37)	Birth cohort, 289 newborns at high risk for asthma and allergic disease development	sIgE antibodies to aeroallergens at the age of 1, 3, 6, and 9 years	6 and 8 years	At the age of 1 year, only sensitization to dog and to cat was significantly associated with asthma risk. At the age of 3 years, sensitization to any perennial allergen was associated with asthma risk
9	Llanora et al. (38)	Cohort study, 78 preschool children 2–5 years with at least one wheezing episode	SPTs to HDM	8–14 years	Children with positive SPT had a twofold higher risk for persistent wheezing at the age 8–14 years
10	Amat et al. (39)	541 infants under 36 months of age who had a history of at least three wheezing episodes	sIgE antibodies to food and inhalant allergens	13 years	Allergen polysensitization (irrespective of the type of allergen), sensitization to multiple aeroallergens and to multiple food allergens were all associated with persistent active asthma
11	Lodge et al. (40)	Birth cohort, 620 infants with a family history of asthma/eczema/allergic rhinitis/severe food allergy	SPTs to food allergens and aeroallergens at the age of 6, 12, and 24 months	12 years	Sensitization to HDM at the age of 12 and 24 months increased the odds for asthma at 12 years
12	Vial Dupuy et al. (41)	200 children who visited a pediatric pulmonology clinic with recurrent wheezing as infants (<2 years)	sIgE antibodies to food allergens and inhalant allergens	6 years	Polysensitization increased the odds for persistent asthma at 6 years of age
13	Caudri et al. (42)	Subpopulation of the PIAMA birth cohort, 848 children who were invited at the age of 3–4 years. For evaluation, they had at least one respiratory symptom suggestive of asthma	sIgE antibodies to inhalant allergens	5–8 years	A positive sIgE to any airborne allergen increased the odds for wheezing at the age of 8 years
14	Lødrup Carlsen et al. (43)	Nested case–control study, 265 children, 2 years old with recurrent (>2 episodes) or persistent (>4 weeks duration) doctor confirmed bronchial obstruction, and 251 controls without bronchial obstruction	sIgE antibodies to food and inhalant allergens	10 years	The probability of current asthma at 10 years of age increased with increasing levels of sIgE antibodies to a mix of allergens measured at 2 years of age. This finding was significant only for boys
15	Simpson et al. (44)	A population-based birth cohort (Manchester Asthma and Allergy Study), 1,186 participants who were recruited at birth and followed at ages 1, 3, 5, and 8 years	sIgE antibodies and SPTs to food and inhalant allergens	8 years	Multiple early atopic sensitization was strongly associated with current wheeze at the age of 8 years. This type of sensitization predicts not only the presence but also the persistence of asthma
16	Marenholz et al. (45)	1,314 children of German MAS birth cohort	sIgE antibodies to food allergens	7, and/or 10, and/or 13 years	Food sensitization increased the odds for asthma
17	Jackson et al. (46)	Birth cohort, 289 newborns with at least one parent with one or more positive aeroallergen SPT and/or a history of physician diagnosed asthma	sIgE to aeroallergens and food allergens at 1 and 3 years of age, plus SPTs to 12 aeroallergens at 5 years of age	6 years of age	Aeroallergen sensitization at the age of 1 and 3 years was associated with increased risk for asthma at the age of 6 years
18	Devulapalli et al. (47)	Nested case–control study Children aged 2 years, 265 cases with recurrent or persistent doctor confirmed bronchial obstruction and 251 controls (the same population as in study No. 14)	SPTs to food allergens and aeroallergens at the age of 2 years	10 years	Atopic sensitization at the age of 2 years did not differ between asthmatic and non-asthmatic children at the age of 10 years
19	Just et al. (48)	A cohort of 219 infants <30 months with recurrent wheezing episodes	sIgE antibodies to aeroallergens and food allergens	6 years	In univariate analysis, allergic sensitization to at least one component of tested allergens was associated with persistence of wheezing at the age of 6 years. Absence of eosinophilia in combination with absence of allergic sensitization discriminated correctly 96% of children with wheezing in remission
20	Piippo-Savolainen et al. (49)	A cohort of 83 children <2 years who were hospitalized for bronchiolitis	sIgE antibodies to aeroallergens	8.5–10 and 13.5–15 years	Early sensitization to seasonal pollens was associated with asthma at the age of 13.5–16 years
21	Eysink et al. (50)	Cohort study, 752 children 1–4 years who had visited GP complaining for cough for at least the preceding 5 days	sIgE antibodies to cat, dog, and HDM	6 years	Sensitization by the age of 4 years was a prognostic indicator of asthma
22	Arshad et al. (51)	Whole population birth cohort in the Isle of Wight, 1,456 newborns	SPTs to aeroallergens and food allergens	10 years	Asthma was associated with positive SPT at the age of 4 years
23	Kotaniemi-Syrjänen et al. (52)	A cohort of 100 infants aged 1–23 months who were hospitalized with infection-related wheezing	sIgE antibodies to aeroallergens and food allergens	5.6–8.8 years	Positive sIgE (>0.35 kU/L) to aeroallergens was associated with the risk of school age asthma. The same was true for sIgE positive test to a mixture of food allergens but with a cut off value of 0.70 kU/L
24	Illi et al. (53)	1,314 children of German MAS birth cohort	sIgE antibodies to food allergens and aeroallergens at 1, 2, 3, 5, 6, and 7 years of age	7 years	Transient sensitization was not a risk factor for asthma at the age of 7 years. Children with persistent sensitization had increased risk for asthma at the age of 7 years provided that there was a positive parental history of asthma or atopy

*SPT, skin prick test; HDM, house dust mite; BHR, bronchial hyperresponsiveness; sIgE, serum immunoglobulin E (IgE); OR, odds ratio; CI: 95% confidence interval*.

Studies referring to population of preschool children were excluded since asthma is not well defined in this age. Definition of current asthma at the age of 6–14 years was not identical among different studies. However, all studies had as a prerequisite evidence of asthma symptoms during the preceding 12 months. It should be also noted that the majority of studies included population at high risk for asthma either because of the parental history or because the population was recruited with respiratory symptoms suggestive of bronchiolitis or wheezing episodes. Despite their heterogeneity, the majority of the presented studies pointed to the same direction, namely, that atopic sensitization of infants or toddlers was associated with increased risk for asthma later in childhood.

Asthma predictive models have also been used in order to assess the probability of a toddler to have asthma as a child. Some of these models included sensitization to allergens in their scoring system. In 2003, Kurukulaaratchy et al. (54) developed a scoring system derived from a birth cohort of Isle of Wight. Although this cohort included all the neonates that were born in 1989 irrespective of their risk level for asthma, the predictive model was a combination of recurrent chest infections at 2 years, atopic sensitization at 4 years, a family history of asthma, and absence of nasal symptoms at 12 months of age. Another predictive model that included atopic sensitization either to at least one aeroallegen or to specific food allergens was the modified asthma predictive index (API) (55), which was derived from the API (56) that was developed in 2000 by Castro-Rodriguez et al. It is, therefore, obvious that atopic sensitization *per se* has not adequate accuracy for the prediction of asthma occurrence in childhood, but it can be used in combination with other risk factors for the prediction of persistence of asthma among preschool wheezers.

It is also of interest to mention that there are studies that investigated if there was a pattern of sensitization that was associated with asthma in childhood. Illi et al. (53) found that transient early sensitization was not associated with an excess risk for asthma in childhood (7 years of age). In cases of persistent atopic sensitization, the risk for childhood asthma increased only in those children with a concomitant maternal history of asthma. However, when sensitization to food allergens was examined separately from that to inhalant allergen, it was shown that early sensitization to food allergens increased the risk for asthma development up to school age. On the contrary, early sensitization to inhalant allergens without concomitant sensitization to food allergens did not confer any excess risk for asthma development up to school age. More recently, Alduraywish et al. (30) showed that early sensitization either to food allergens or to aeroallergens was associated with increased risk for asthma in childhood. However, the strongest effect on asthma risk was observed in children with concurrent sensitization to food and inhalant allergens. In the latter study (30), there are no data regarding persistence of sensitization as available information is restricted to the age of 24 months. On the other hand, Simpson et al. (57) using a similar approach found that multiple early sensitization has the strongest association with asthma at the age of 8 years. However, they concluded that IgE antibody responses do not represent a single phenotype of atopy, but rather, several different atopic vulnerabilities, which vary in their relation with asthma presence and severity. In their study, there were no data regarding parental history of asthma or atopy and, so, it is not possible to make direct comparisons with Illi et al.’s findings.

A different pattern of atopic sensitization in relation to childhood asthma risk was identified by Stoltz et al. (37). In their birth cohort study, only sensitization to dog and cat at the first year of life or sensitization to any of the perennial allergens tested was associated with asthma risk at the age of 6 years.

Although it is well recognized that atopy does not equate to atopic sensitization (58), it seems that the latter serves as a good surrogate of atopy. Most likely, the latter constitutes a risk factor for asthma in childhood only when other prerequisites coexist such as history of infant wheezing, parental history of asthma, or parental history of atopy. Therefore, it cannot be considered a satisfactory indicator for predicting or ruling out asthma in childhood without first taking into account these factors. This comes as no surprise since asthma is a multifactorial entity dependent on genetic predispositions and environmental exposures. Based on the available evidence, wheezy toddlers with a positive family history of asthma and presence of atopic sensitization should be regarded as high risk for persistence of asthma in childhood. Similarly, in a recent systematic review, Rotriguez-Martinez et al. (59) included allergic sensitization early in life (especially polysensitization) among the factors that confer to the prediction of wheezing persistence through school age.

## The Role of Allergy in the Prevention of Asthma

Predicting the risk of childhood asthma development or persistence would be of great importance as it could facilitate the design of primary prevention measures. The last decade of the previous century, it was hypothesized that primary prevention strategies in terms of allergen avoidance for infants at high risk for asthma would be of benefit. Nowadays, it has been recognized that prevention of asthma is not so straightforward as it seemed due to the multifactorial nature of the disease.

Many different interventional studies have been conducted over the last 20 years that tested the efficacy of allergen avoidance in the prevention of asthma. A recent meta-analysis (60) examined the mono- and multifaceted intervention studies until 2011. They found that monofaceted intervention studies versus control did not lead to any reduction in childhood asthma occurrence. In contrast, multifaceted intervention studies were associated with a reduction by half of asthma diagnosis in childhood. Three intervention studies were characterized as multifaceted and were included in this meta-analysis and in two of them the asthma outcome was reported at an age older than 6 years (51, 61). Both studies included interventions regarding reduction of house dust mite and food exposure, whereas the Canadian study (61) also included measures for pet avoidance and reduction in environmental tobacco smoke. Both studies included children at high risk for developing asthma and, therefore, their results are not applicable in the general population. Furthermore, taking into consideration that asthma risk attributed to atopy varied substantially between affluent and non-affluent countries, the same intervention measures may not be of value in other population settings even in children at high risk for asthma.

The outcome is noteworthy not only for the ineffectivenes of the monofaceted interventions but also because it generated a new hypothesis that had to be tested: that early exposure to specific allergens may lead to tolerance instead of allergy. It was shown (62) that chronic exposure to high concentrations of cat allergens can promote a modified immune response consisting of a reduction in specific IgE immunoglobulins and an increase in specific IgG immunoglobulins, especially IgG4. Similarly, Stoltz et al. (37) found that although sensitization to dog was associated with increased asthma risk, dog exposure at birth was associated with a reduced asthma risk irrespective of sensitization status to dog, at any point during the first 6 years of life. The aspect of tolerance development through early exposure has also been evident in the area of food allergy. Tolerance to peanuts was developed in infants through the early introduction of peanut in their diet instead of strict avoidance whereas high maternal consumption of peanuts during pregnancy reduced the risk of peanut allergy in the offspring (63). These observations goes in parallel with the results of a recent meta-analysis (64), which showed that maternal dietary restriction during pregnancy or lactation failed to reduce the child’s risk for atopic disease. There is no evidence that any modification of the weaning procedure could help in the prevention of atopic diseases, and so, such interventions are not recommended (65).

Having taken into account all recent evidence, the German updated guidelines on allergy prevention, published in 2014 (66), do not recommend any restriction in maternal diet during pregnancy and during lactation. They also do not recommend any measures for the reduction of house dust mite exposure for primary prevention purposes. However, they recommended avoidance of exposure to tobacco smoke and other indoor air pollutants as well as indoor conditions that promote mold. They also recommended some other measures of prevention that seem to affect the risk of asthma such as avoidance of obesity and avoidance of cesarean section.

More recently, in an international report about the implications of indoor allergen avoidance in asthma prevention and management, it was also acknowledged that the results for HDM avoidance and asthma prevention were inconsistent. The authors attributed this discrepancy to the type of approach (multifaceted or monofaceted), to the initial level of indoor HDM allergen, and possibly to genetic or other environmental co-factors (67).

Therefore, based on the existing evidence, allergen avoidance cannot confer sufficiently to the primary prevention of asthma in childhood. Allergen exposure may increase the risk of atopic consequences, but the outcome is highly dependent on the dose and kind of allergen, timing of exposure, and vulnerability of the child (68). Nevertheless, it is of benefit to identify those infants who are at increased risk for the development of asthma in order to (a) intensify the implementation of measures of primary prevention according to the existing guidelines (66) and (b) provide them the best practice management, close clinical monitoring, and measures of secondary prevention.

## Conclusion

Atopic sensitization is related with an increased risk for asthma in childhood, but the extent of this association varies considerably among different populations and there is a pronounced difference between affluent and non-affluent countries; strong relations are observed mostly in affluent Western countries, whereas in less affluent countries, a more heterogeneous picture is observed.

Atopic sensitization *per se* does not predict development or persistence of asthma in childhood although it seems that atopic sensitization is associated with development of asthma in patients with wheezing.

Although allergen avoidance had been considered as a potential preventive measure from asthma development in high risk children, the majority of intervention studies failed to show positive results.

The identification, however, of infants at high risk for the development or persistence of asthma in childhood, it remains of benefit as these subjects should be provided with the best practice management.

## Author Contributions

MM, co-designed the structure of the paper, wrote most of the manuscript, reviewed, and approved the final manuscript. KD: co-designed the structure of the paper, wrote some parts of the manuscript, reviewed, corrected, and approved the final manuscript. IL and ST: searched PubMed and libraries and found the available literature and wrote some parts of the manuscript.

## Conflict of Interest Statement

The authors declare that the research was conducted in the absence of any commercial or financial relationships that could be construed as a potential conflict of interest.
